# Micro-epidemiology and spatial heterogeneity of *P*. *vivax* parasitaemia in riverine communities of the Peruvian Amazon: A multilevel analysis

**DOI:** 10.1038/s41598-017-07818-0

**Published:** 2017-08-14

**Authors:** Gabriel Carrasco-Escobar, Dionicia Gamboa, Marcia C. Castro, Shrikant I. Bangdiwala, Hugo Rodriguez, Juan Contreras-Mancilla, Freddy Alava, Niko Speybroeck, Andres G. Lescano, Joseph M. Vinetz, Angel Rosas-Aguirre, Alejandro Llanos-Cuentas

**Affiliations:** 1 Laboratorio ICEMR-Amazonia, Laboratorios de Investigacióny Desarrollo, Facultad de Cienciasy Filosofia, Universidad Peruana Cayetano Heredia, Lima, Peru; 2 Facultad de Salud Públicay Administración, Universidad Peruana Cayetano Heredia, Lima, Peru; 3 Instituto de Medicina Tropical “Alexander von Humboldt”, Universidad Peruana Cayetano Heredia, Lima, Peru; 4 Departamento de Ciencias Celularesy Moleculares, Facultad de Ciencias y Filosofia, Universidad Peruana Cayetano Heredia, Lima, Peru; 5 Department of Global Health and Population, Harvard T.H. Chan School of Public Health, Boston, MA USA; 6 Department of Biostatistics, University of North Carolina Gillings School of Global Public Health, Chapel Hill, NC USA; 7 Department of Health Research Methods, Evidence and Impact, McMaster University, Hamilton, ON Canada; 8 Región de Salud Loreto, Iquitos, Loreto, Peru; 9 Research Institute of Health and Society (IRSS), Université Catholique de Louvain, Brussels, Belgium; 10 Division of Infectious Diseases, Department of Medicine, University of California San Diego School of Medicine, La Jolla, California USA

## Abstract

Malaria has steadily increased in the Peruvian Amazon over the last five years. This study aimed to determine the parasite prevalence and micro-geographical heterogeneity of *Plasmodium vivax* parasitaemia in communities of the Peruvian Amazon. Four cross-sectional active case detection surveys were conducted between May and July 2015 in four riverine communities in Mazan district. Analysis of 2785 samples of 820 individuals nested within 154 households for *Plasmodium* parasitaemia was carried out using light microscopy and qPCR. The spatio-temporal distribution of *Plasmodium* parasitaemia, dominated by *P*. *vivax*, was shown to cluster at both household and community levels. Of enrolled individuals, 47% had at least one *P*. *vivax* parasitaemia and 10% *P*. *falciparum*, by qPCR, both of which were predominantly sub-microscopic and asymptomatic. Spatial analysis detected significant clustering in three communities. Our findings showed that communities at small-to-moderate spatial scales differed in *P*. *vivax* parasite prevalence, and multilevel Poisson regression models showed that such differences were influenced by factors such as age, education, and location of households within high-risk clusters, as well as factors linked to a local micro-geographic context, such as travel and occupation. Complex transmission patterns were found to be related to human mobility among communities in the same micro-basin.

## Introduction

Progress in reducing the malaria burden in the last decade has been substantial worldwide, yet key challenges remain, particularly with a global emphasis on malaria elimination^1, 2^. According to data after World War II, malaria transmission in Peru has been dominated by *Plasmodium vivax*. Since the 1990s *P*. *falciparum*, in addition to *P*. *vivax*, emerged as a major epidemic, with continued outbreaks and rising incidence continuing to the present^3–6^. In the last decades, five years (2005–2010) of funding from the Global Fund for AIDS, TB and Malaria (Pan-Andean Program for Malaria Control in the Border – PAMAFRO) was used to scale-up comprehensive control strategies, which included improving early diagnosis and treatment of malaria cases in endemic areas, strengthening of health services systems, training health promoters and microscopists, distributing insecticide-treated bed nets and environmental management through community participation^6–8^. As a result, the number of passively reported cases of malaria in 2010 was the lowest in the previous two decades (11,604 cases).

Since then, Malaria control activities in the Iquitos region fall within the purview of the Regional Health Directorate of Loreto (RHDL), under the auspices of the national Ministry of Health (MOH). As a matter of operational policy, passive and reactive case detection is based on light microscopy which then determines treatment of confirmed infections. The increased numbers of malaria cases reported since 2011 in Loreto department are due to a variety of factors: expansion of residual foci of transmission with new infections; reintroductions from *P*. *vivax* relapse, change in *Anopheles darlingi* populations and behaviours (the primary malaria vector in Amazonia)^9–12^, sub-microscopic infection reservoirs^13, 14^, general weather-related factors (such as flooding in Loreto in recent years) and reduced access of rural dwellers to health services^3, 15^.

Located in the Amazon Region, Loreto department covers 28.7% (368,851 Km^2^) of total Peruvian territory, encompassing 3.3% (1,018,160 hab.) of the total population of Peru^16^, and 95% of the countrywide malaria burden. Despite the fact that malaria transmission is perennial in the Peruvian Amazon basin, with a high transmission season between November and June^5^, RHDL data suggest that Loreto has a heterogeneous spatio-temporal malaria distribution^3^.

In this context, it is sometimes possible to distinguish groups with greater malaria risk^17^. Spatially, infections aggregated at the household-level may reveal clusters of households in which the risk of a malaria infection is higher in comparison with the rest of the community^18–21^. The detection of these high-risk clusters can help target malaria control efforts^18, 22–24^. More importantly, from the perspective of public health control measures, clusters should be identified regardless of the symptomatology or parasite density of the carriers, since the acquired immunity of the high-risk clusters inhabitants^25–29^, and the clonality of malaria parasites in the Amazon Region^30–34^, generates a higher proportion of undetectable infections (sub-microscopic) that would not otherwise come for treatment due to lack of symptoms. In this scenario, it would be expected that different spatial aggregation patterns would be identified for malaria disease (that is typically, microscopic parasitaemia accompanied by symptoms) and malaria parasitaemia (with or without symptoms)^19^.

The particular biology of *P*. *vivax* includes a dormant liver stage (i.e. hypnozoites) that remains undetectable until unknown mechanisms^35^ reactivate the parasite cycle and cause relapses from the original infection^36^. Importantly, the treatment of *P*. *vivax* in Peru was based on a regimen of 7 days of primaquine (PQ) at 0.5 mg/kg/day. However this regimen is not typically supervised and an adherence rate of only 62% has previously been reported^37^. Tools to distinguish between new infections and hypnozoite relapses are still in development^38–41^; therefore, here the term parasitaemia refers to indicate the presence of *Plasmodium* parasites in the blood, as suggested by the World Health Organization^42^, regardless of its origin from new infection or hypnozoite relapse^43^.

Previous studies in communities near Iquitos (the capital of Loreto department) show spatial and micro-epidemiological heterogeneity of malaria transmission both in terms of incidence of disease and parasite prevalence^14, 44, 45^. Nonetheless, the complex dynamics of malaria transmission in riverine communities – the typical malaria context in Amazonia – require accurate identification of these high-risk clusters of *P*. *vivax* parasitaemia and the associated factors at the person, household and community levels, to improve the implementation of intervention strategies in these foci of infections^22, 46, 47^. This study aimed to determine the parasite prevalence and the heterogeneity of risk factors for *P*. *vivax* parasitaemia between communities, taking into account the clustering of parasitaemia within households and within communities. Thus, a spatial and multilevel analysis was carried out using spatial information in four riverine communities of the Peruvian Amazon Region.

## Results

### Socio-demographic and household characteristics

**Figure 1:**
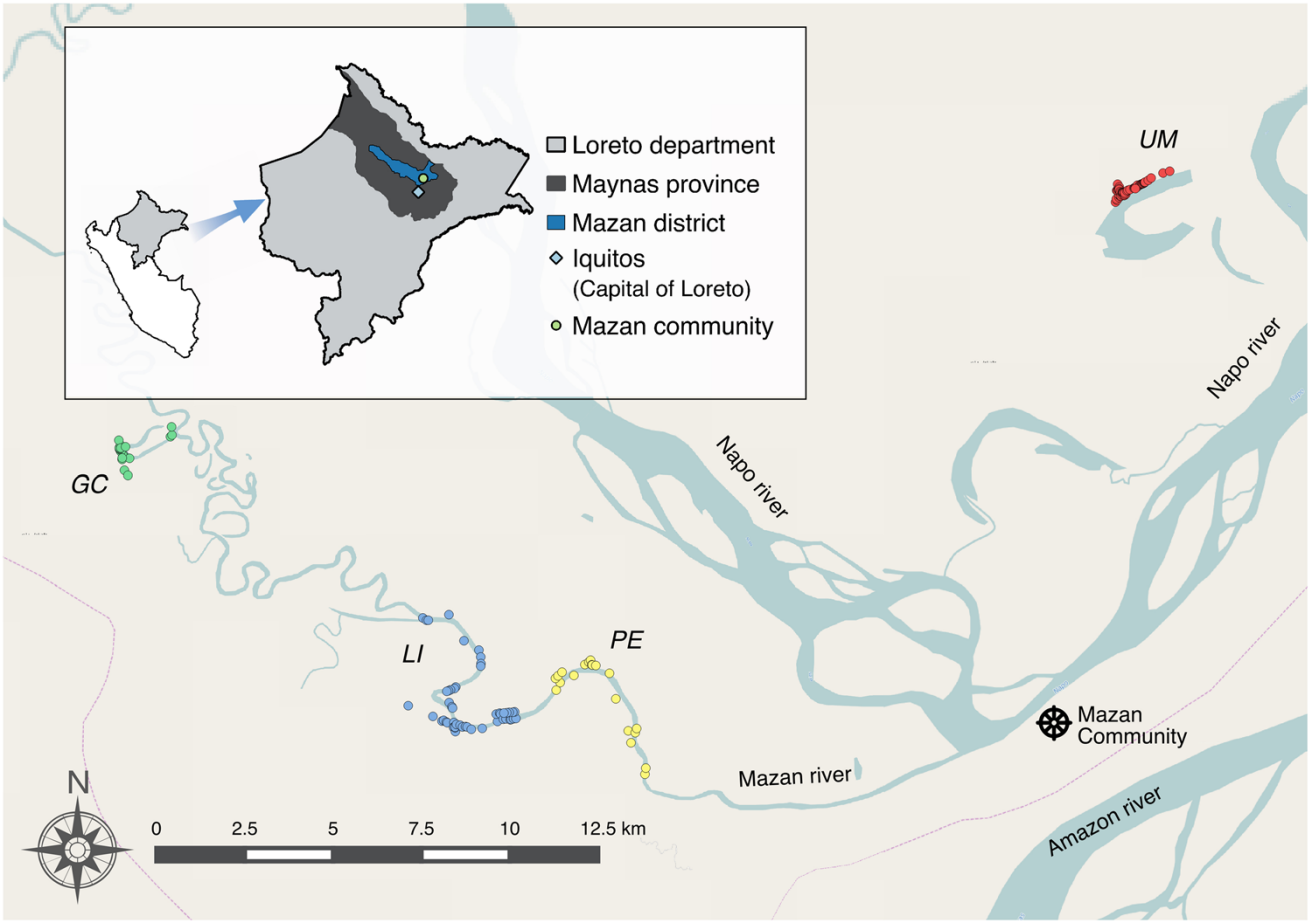
Study area in Mazán district, Loreto Department, Peruvian Amazon. Spatial distribution of Gamitanacocha (GC, green circles), Libertad (LI, blue circles), Primero de Enero (PE, yellow circles) and Urco Miraño (UM, red circles) communities in the Mazan district. Map generated with QGIS 2.16 (QGIS Development Team, 2016. QGIS Geographic Information System. Open Source Geospatial Foundation Project. http://www.qgis.org/).

Of the 935 censused inhabitants in the four communities, a total of 820 individuals (Ind) from 154 households (HHs) were included in the analysis (Fig. 1); three individuals were excluded due to lack of qPCR test during follow-up. Overall, 52.4% of participants were male, and 48.8% were under 15 years old (Table 1). The majority of individuals had primary school level education (complete or incomplete) representing between 43.2% and 61.6% of the participants across communities. Overall, the most common occupation was subsistence farming (37.5%) and the main staple crops grown across the four communities were cassava and banana. One out of ten inhabitants (11.7%) self-reported travel outside the community in a period of one month (Table 1) and the most common destinations of the 261 travel records are presented in Supplementary Table S1.

**Table 1:** Baseline characteristics of the study population.

Characteristics	% Individuals					% Households				
	GC	LI	PE	UM	Total	GC	LI	PE	UM	Total
	n = 92 (%)	n = 298 (%)	n = 99 (%)	n = 331 (%)	n = 820 (%)	N = 19 (%)	N = 56 (%)	N = 20 (%)	N = 59 (%)	N = 154 (%)
**Time-dependent Level variables**										
***P***. ***vivax*** **parasitaemia by PCR**										
Positive	27 (29.4)	151 (50.7)	38 (38.4)	170 (51.4)	386 (47.1)	15 (78.9)	49 (87.5)	16 (80.0)	52 (88.1)	132 (85.7)
Negative	65 (70.6)	147 (49.3)	61 (61.6)	161 (48.6)	434 (52.9)	4 (21.1)	7 (12.5)	4 (20.0)	7 (11.9)	22 (14.3)
**Fever symptom**										
Positive	18 (19.6)	34 (11.4)	4 (4.0)	46 (13.9)	102 (12.4)	10 (52.6)	17 (30.4)	3 (15.0)	24 (40.7)	54 (35.1)
Negative	74 (80.4)	264 (88.6)	95 (96.0)	285 (86.1)	718 (87.6)	9 (47.4)	39 (69.6)	17 (85.0)	35 (59.3)	100 (64.9)
**Travel record in the last month**										
Positive	18 (19.6)	33 (11.1)	20 (20.2)	25 (7.5)	96 (11.7)	11 (57.9)	20 (35.7)	9 (45.0)	16 (27.1)	56 (36.4)
Negative	74 (80.4)	265 (88.9)	79 (79.8)	306 (92.5)	724 (88.3)	8 (42.1)	36 (64.3)	11 (55.0)	43 (72.9)	98 (63.6)
**Individual Level variables**										
**Gender**										
Male	43 (46.7)	159 (53.4)	54 (54.5)	174 (52.5)	430 (52.4)	18 (94.7)	54 (96.4)	18 (90.0)	59 (100.0)	149 (96.7)
Female	49 (53.3)	139 (46.6)	45 (45.4)	157 (47.4)	390 (47.6)	1 (5.3)	2 (3.6)	2 (10.0)	0 (00.0)	5 (3.3)
**Age groups** (**years**)										
<15	41 (44.6)	132 (44.3)	49 (49.5)	178 (53.8)	400 (48.8)	15 (78.9)	41 (73.2)	16 (80.0)	50 (84.7)	122 (79.2)
≥15	51 (55.4)	166 (55.7)	50 (50.5)	153 (46.2)	420 (51.2)	4 (21.1)	15 (26.8)	4 (20.0)	9 (15.3)	32 (20.8)
**Education level**										
None or primary school	25 (27.2)	98 (32.9)	26 (26.3)	94 (28.4)	243 (29.6)	14 (73.7)	45 (80.4)	16 (80.0)	44 (74.6)	119 (77.3)
Secondary school or higher	67 (72.8)	200 (67.1)	73 (73.7)	237 (71.6)	577 (70.4)	5 (26.3)	11 (19.6)	4 (20.0)	15 (25.4)	35 (22.7)
**Outdoor Occupation**										
(**Logger**, **Fisher and Farmer**)										
Yes	47 (51.1)	140 (47.0)	42 (42.4)	104 (31.4)	333 (40.6)	18 (94.7)	53 (94.6)	19 (95.0)	49 (83.0)	139 (90.3)
No	45 (48.9)	158 (53.0)	57 (57.6)	227 (68.6)	487 (59.4)	1 (5.3)	3 (5.4)	1 (5.0)	10 (17.0)	15 (9.7)
**Household Level variables**										
**High-risk cluster location°**										
Yes	72 (78.3)	0 (0.0)	25 (25.2)	199 (60.1)	296 (36.1)	14 (73.7)	0 (0.0)	5 (25.0)	35 (59.3)	54 (35.1)
No	20 (21.7)	298 (100.0)	74 (74.7)	132 (39.9)	524 (63.9)	5 (26.3)	56 (100.0)	15 (75.0)	24 (40.7)	100 (64.9)
**Household Structure**										
Open Houses	34 (37.4)	107 (36.4)	57 (57.6)	115 (34.7)	313 (38.4)	8 (44.4)	21 (38.2)	11 (55.0)	20 (33.9)	60 (39.5)
At least one closed room	57 (62.6)	187 (63.6)	42 (42.4)	216 (65.3)	502 (61.6)	10 (55.6)	34 (61.8)	9 (45.0)	39 (66.1)	92 (60.5)
**Predominant material in walls**										
Cement or Wood	70 (76.1)	256 (85.9)	77 (77.8)	244 (73.7)	647 (78.9)	15 (79.0)	48 (85.7)	16 (80.0)	42 (71.2)	121 (78.6)
Palm, leaf, straw, corrugated plastic	22 (23.9)	42 (14.1)	22 (22.2)	87 (26.3)	173 (21.1)	4 (21.0)	8 (14.3)	4 (20.0)	17 (28.8)	33 (21.4)
**Predominant material in roof** ^†^										
Corrugated plastic	31 (34.1)	12 (4.1)	0 (0.0)	141 (42.6)	184 (22.6)	5 (27.8)	5 (9.1)	0 (0.0)	24 (40.7)	34 (22.4)
Palm, leaf, straw	60 (65.9)	282 (95.9)	99 (100.0)	190 (57.4)	631 (77.4)	13 (72.2)	50 (90.9)	20 (100.0)	35 (59.3)	118 (77.6)
**Household sprayed** ^*,†^										
Yes	77 (84.6)	266 (90.5)	89 (89.9)	325 (99.7)	757 (93.5)	15 (83.3)	47 (85.5)	18 (90.0)	56 (98.3)	136 (90.7)
No	14 (15.4)	28 (9.5)	10 (10.1)	1 (0.3)	53 (6.5)	3 (16.7)	8 (14.5)	2 (10.0)	1 (1.7)	14 (9.3)
**Electricity supply**										
Yes	24 (26.4)	80 (27.2)	31 (31.3)	269 (81.3)	404 (49.6)	4 (22.2)	13 (23.6)	5 (25.0)	45 (76.3)	67 (44.1)
No	67 (73.6)	214 (72.8)	69 (69.7)	62 (18.7)	411 (50.4)	14 (77.8)	42 (76.4)	15 (75.0)	14 (23.7)	85 (55.9)
**Livestock inside dwelling** ^†,+^										
Yes	24 (26.4)	107 (36.4)	43 (43.4)	107 (32.3)	281 (34.5)	5 (27.8)	22 (40.0)	8 (40.0)	17 (28.8)	52 (34.2)
No	67 (73.6)	187 (63.6)	56 (56.6)	224 (67.7)	534 (65.5)	13 (72.2)	33 (60.0)	12 (60.0)	42 (71.2)	100 (65.8)

Proportions were expressed at a) individual level, where time-dependent variables were collapsed by calling it positive if there was at least one positive event at any of the time points (4 ACD surveys) and b) household level, where individual-dependent variables were collapsed based on whether at least one individual in the household had the reference characteristic. GC = Gamitanacocha; LI = Libertad; PE = Primero de Enero; UM = Urco Miraño; ^†^Variables with missing values; °Including 550m-neighborhood buffer; *Indoor Residual Spraying; ^+^Chicken, cows, dog or cats.

The sample was distributed over four communities as follows: two large communities, Libertad (56 HHs, 298 Ind) and Urco Miraño (59 HHs, 331 Ind); and two smaller communities, Gamitanacocha (19 HHs, 92 Ind) and Primero de Enero (20 HHs, 99 Ind). Of all communities, 60 households (39.5%) had an open structure (no walls to complete at least one closed room), with higher proportions in Gamitanacocha and Primero de Enero, than in Libertad and Urco Miraño. The predominant materials in walls were cement or wood (78.6% of HHs) and in roofs were palm, leaf or straw (77.6% of HHs). Approximately one quarter of the households in each community had an electricity supply, except Urco Miraño where three-fourths (76.3%) of HHs used solar panels or diesel-powered generators. Inhabitants of most households (90.7%) reported that the household received indoor residual spraying (IRS) by the RHDL in the last five years, and one-third (34.2%) of households had livestock inside the dwelling.

### Malaria parasite prevalence

**Figure 2:**
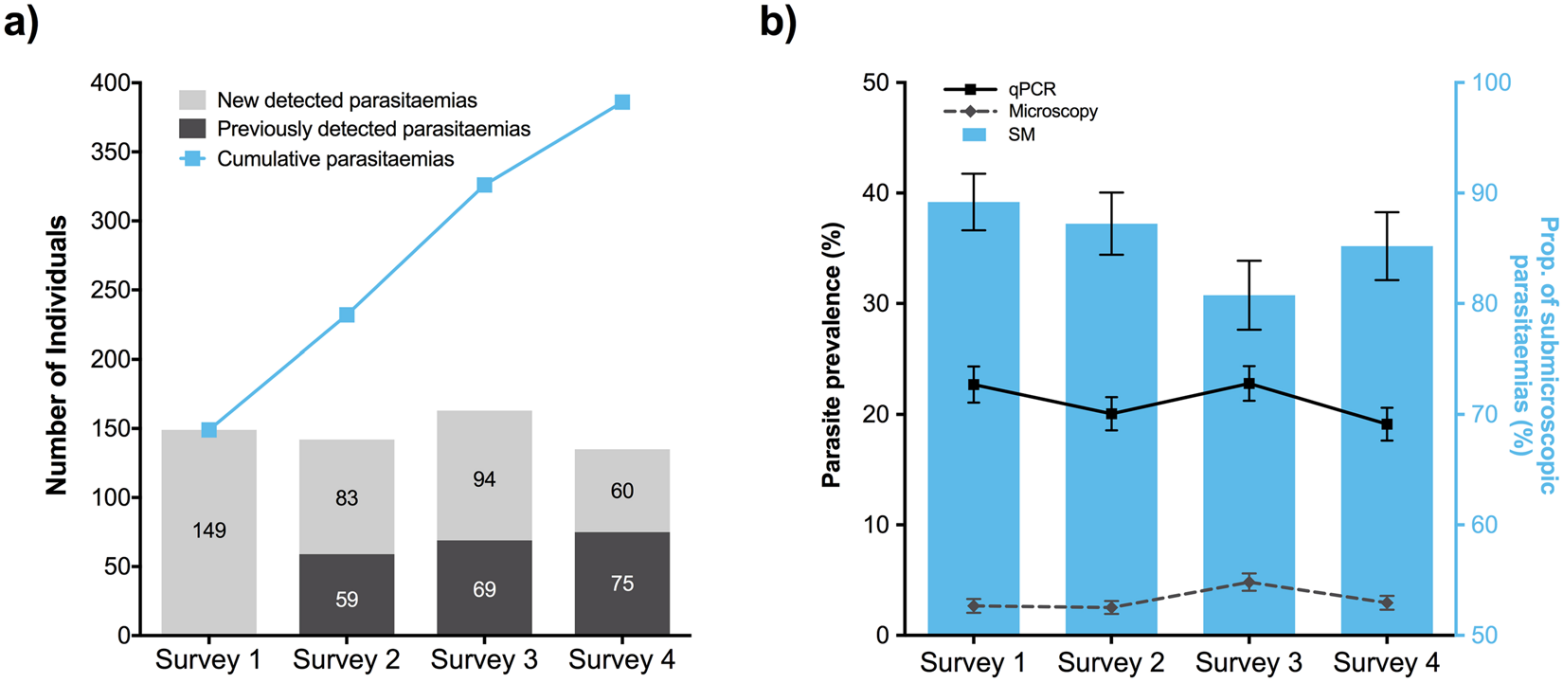
Distribution of *P*. *vivax* parasitaemia and parasite prevalence over time (4 ACD surveys): (**a**) Number individuals with new detected (light grey bars), previously detected (dark grey bars) and cumulative (light blue squares) *P*. *vivax* parasitaemia, and (**b**) *P*. *vivax* parasite prevalence by microscopy (grey dashed line), and qPCR (black solid line), and the proportion of submicroscopic parasitaemia (SM, light blue bars) in each survey round. Error bars represent the standard error of the proportion (SEP) in each survey round. Surveys were conducted from May to July 2015, with 10 days between surveys.

Throughout the four ACD surveys, 515 inhabitants had samples drawn at four visits, 172 at three visits, 76 at two visits and 57 at one visit. In total, 2,785 blood samples were examined for *Plasmodium spp*. parasitaemia by qPCR and 2,768 slides (99.4% of samples collected) were examined by light microscopy. The cumulative number of individuals with *P*. *vivax* parasitaemia over the study period and the *P*. *vivax* parasite prevalence detected by light microscopy and qPCR in each survey round are presented in Fig. 2.

Of the total of blood spots tested, 711 (25.5%) were positive for *Plasmodium spp*.*;* 589 *P*. *vivax* and 122 *P*. *falciparum*. In contrast, 95 (3.4%) of the available slides were positive for *Plasmodium spp*.; 89 *P*. *vivax* and 6 *P*. *falciparum* (Table 2). Four blood samples positive to *P*. *vivax* by qPCR were not available for light microscopy. Of the remaining 707 qPCR-positive samples, 616 (87.1%) were light microscopy negative, i.e. sub-microscopic parasitaemia (there were slight variations between surveys) (Fig. 2b). The proportion of sub-microscopic parasitaemia was higher in *P*. *falciparum* (95.1%; 116/122) than in *P*. *vivax* (85.5%; 500/585). The sensitivity and specificity of light microscopy using qPCR as the reference method was 12.9% (95% CI = 10.5%;15.6%) and 99.8% (95% CI = 99.5%;99.9%), respectively. However, qPCR did not detect 4 *Plasmodium spp*. parasitaemia (3 *P*. *vivax* and 1 *P*. *falciparum*) that were identified by light microscopy.

**Table 2:** Malaria parasite prevalence (by microscopy and PCR) by blood samples and individuals.

Characteristics	Gamitanacocha		Libertad		Primero de Enero		Urco Miraño		Total	
	Samp. n = 265 (%)	Ind.^ n = 92 (%)	Samp. n = 986 (%)	Ind.^ n = 298 (%)	Samp. n = 330 (%)	Ind.^ n = 99 (%)	Ind.^ n = 1204 (%)	Samp. n = 331 (%)	Ind.^ n = 2785 (%)	Samp. n = 820 (%)
***Plasmodium spp***. **parasitaemia by qPCR**										
Positive	48 (18.1)	34 (37.0)	314 (31.8)	183 (61.4)	67 (20.3)	44 (44.4)	282 (23.4)	190 (57.4)	711 (25.5)	451 (55.0)
Negative	217 (81.9)	58 (63.0)	672 (68.2)	115 (38.6)	263 (79.7)	55 (55.6)	922 (76.6)	141 (42.6)	2074 (74.5)	369 (45.0)
***P***. ***vivax*** **parasitaemia by qPCR**										
Positive	39 (14.7)	27 (29.4)	236 (23.9)	151 (50.7)	59 (17.9)	38 (38.4)	255 (21.2)	170 (51.4)	589 (21.1)	386 (47.1)
Negative	226 (85.3)	65 (70.6)	750 (76.1)	147 (49.3)	271 (82.1)	61 (61.6)	949 (78.8)	161 (48.6)	2196 (78.9)	434 (52.9)
***P***. ***falciparum*** **parasitaemia by qPCR**										
Positive	9 (3.4)	8 (8.7)	78 (7.9)	43 (14.4)	8 (2.4)	7 (7.1)	27 (2.2)	27 (8.2)	122 (4.4)	85 (10.4)
Negative	256 (96.6)	84 (91.3)	908 (92.1)	255 (85.6)	322 (97.6)	92 (92.9)	1177 (97.76)	304 (91.8)	2663 (95.6)	735 (89.6)
**LM result** ^†^										
*P*. *falciparum*	0 (0.0)	0 (0.0)	6 (0..6)	6 (2.0)	0 (0.0)	0 (0.0)	0 (0.0)	0 (0.0)	6 (0.2)	6 (0.7)
*P*. *vivax*	1 (0.4)	1 (1.1)	24 (2.4)	24 (8.1)	13 (4.0)	11 (11.1)	51 (4.2)	50 (15.1)	89 (3.2)	86 (10.5)
Negative	263 (99.6)	91 (98.9)	949 (96.9)	266 (89.9)	311 (96.0)	88 (88.9)	1150 (95.8)	281 (84.9)	2673 (96.6)	726 (88.8)

Samp. = Samples; Ind. = Individuals; LM = Light microscopy; ^^^At least in one positive in the four surveys; ^†^Variables with missing values.

From the 820 participants that were tested by qPCR at least in one ACD survey, 386 (47.1%) had at least one *P*. *vivax* and 85 (10.4%) *P*. *falciparum* parasitaemia during the study period. In the surveys, qPCR melting analysis did not identify mixed species parasitaemia, probably because the subjects in the active surveillance screens were predominantly asymptomatic (less than 13% symptomatic; Table 1), hence with very low parasite densities. We did find that 20 subjects (2.4% of total participants) had *P*. *vivax* or *P*. *falciparum* at different times. The parasite prevalence of *Plasmodium spp*. by qPCR across the four surveys ranged from 23.9% to 27.1% with a participation rate >80% in all survey rounds.

The overall cumulative parasite prevalence, meaning the proportion of individuals with at least one *Plasmodium spp*. parasitaemia by qPCR across location and time (survey rounds), was 55% (451/820). By light microscopy, only 86 (10.5%) participants had *P*. *vivax* and 6 (0.7%) had *P*. *falciparum* parasitaemia (Table 2). The proportion of *P*. *vivax* parasitaemia detected by qPCR (44.6–49.5%) and light microscopy (1–16.5%) decreases in older people. By contrast, the proportion of *P*. *falciparum* parasitaemia by qPCR (9.2–14.1%) and light microscopy (0.7–1.2%) increases across age.

### Spatial clustering of *P. vivax* parasitaemia in households

**Figure 3:**
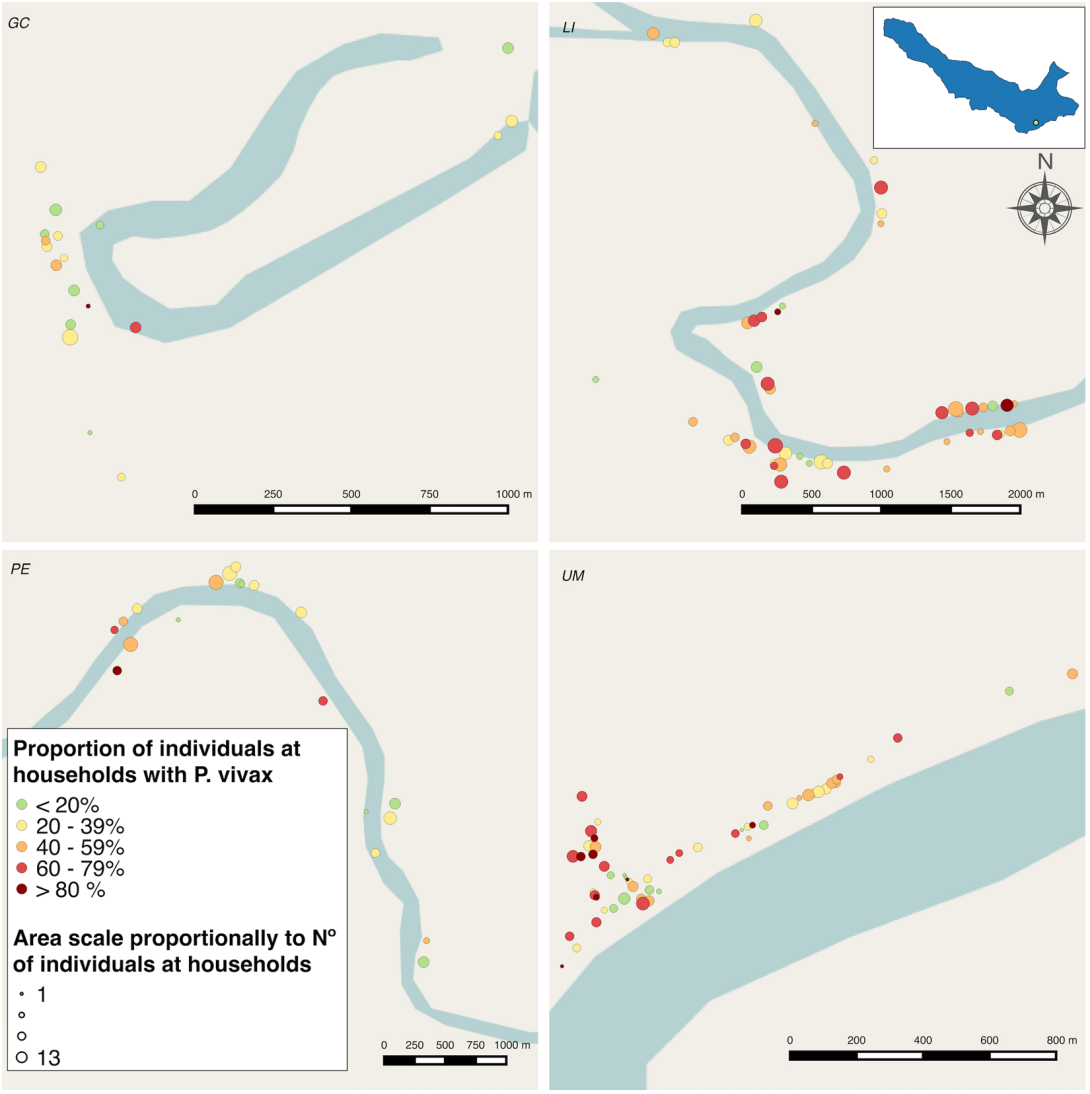
Proportion of participants per household with at least one *P*. *vivax* parasitaemia along the follow-up in: Gamitanacocha (GC), Libertad (LI), Primero de Enero (PE) and Urco Miraño (UM). Each point represents a household location. Map generated with QGIS 2.16 (QGIS Development Team, 2016. QGIS Geographic Information System. Open Source Geospatial Foundation Project. http://www.qgis.org/).

Nearly all households (90.3%) had at least one participant with a *Plasmodium spp*. parasitaemia during the study, and 85.7% and 39.0% had ≥1 participants with at least one *P*. *vivax* or *P*. *falciparum* parasitaemia in the follow-up, respectively. Given the small number of *P*. *falciparum* qPCR-positive parasitaemia, spatial and risk factors analyses were carried out only for *P*. *vivax* parasitaemia and *P*. *falciparum* parasitaemia were considered as negative for *P*. *vivax*. The household-level spatial distribution of individuals with at least one *P*. *vivax* parasitaemia during the study period is presented in Fig. 3.

**Figure 4:**
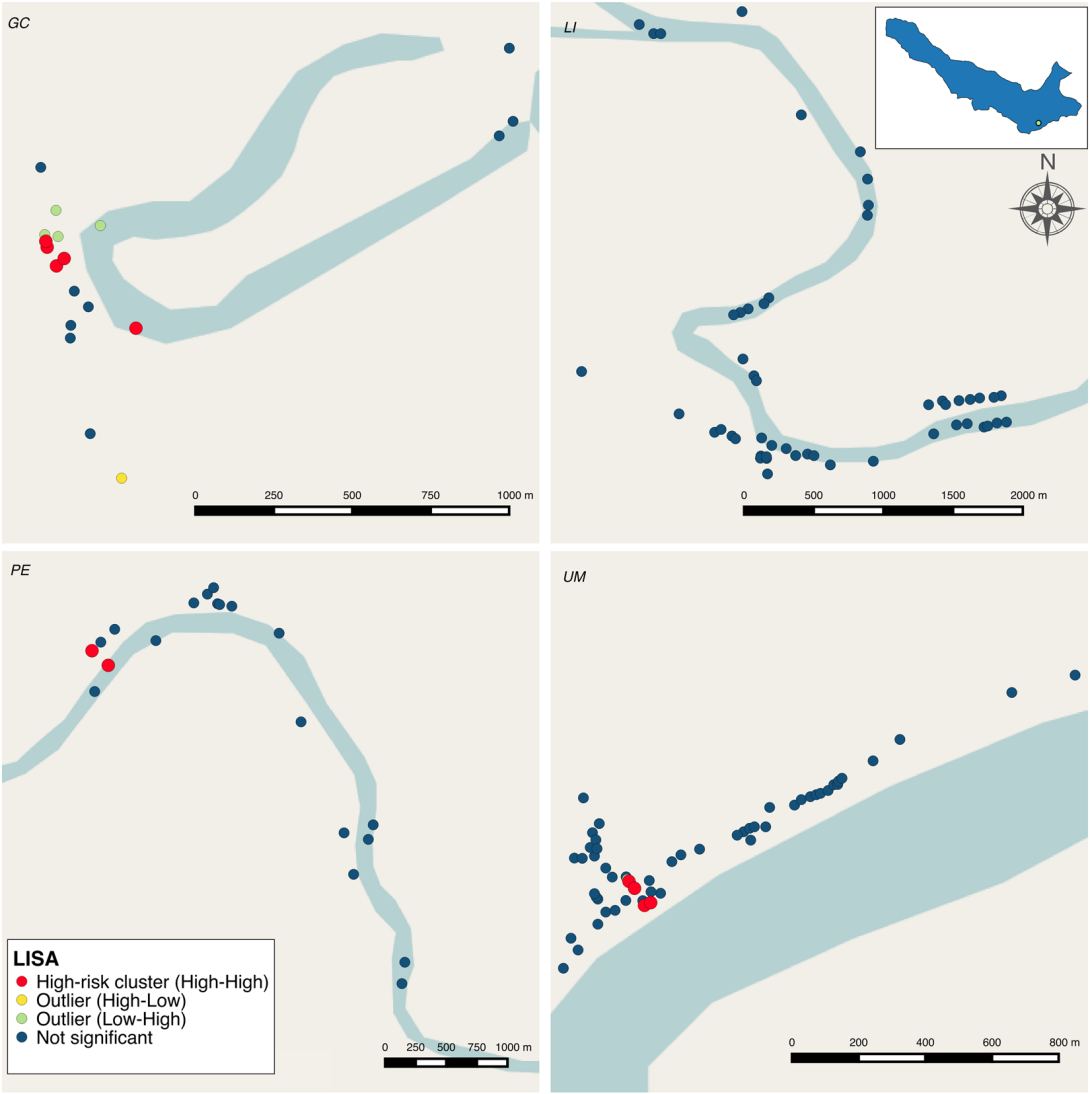
Local Indicators of Spatial Association (LISA) clustering analysis of *P*. *vivax* parasitaemia in: Gamitanacocha (*GC*), Libertad (*LI*), Primero de Enero (*PE*) and Urco Miraño (*UM*). Each point represents a household location. Statistically significant clusters corrected by false discovery rate were represented as follows: Households with high parasitaemia surrounded by a neighborhood with high parasitaemia (High-risk cluster − red circles), households with high parasitaemia surrounded by a neighborhood with low parasitaemia (High-low outlier − yellow circles) and households with low parasitaemia surrounded by a neighborhood with high parasitaemia (Low-high outlier − green circles). Map generated with QGIS 2.16 (QGIS Development Team, 2016. QGIS Geographic Information System. Open Source Geospatial Foundation Project. http://www.qgis.org/).

The global spatial analysis showed the presence of positive spatial autocorrelation only in Primero de Enero (global Moran’s *I* = 0.26; p-value = 0.04) while in other communities, global Moran’s *I* values were statistically not significant. FDR-corrected LISA analysis detected statistically significant clusters in three of the four communities (Fig. 4). High-risk clusters (and their neighborhoods) were considerably bigger in Gamitanacocha and Urco Miraño than in Primero de Enero. In Gamitanacocha, the high-risk cluster comprised 5 households (26.3% of households) encompassing one quarter of the community population (LISA range = 0.03–0.56; p-value range = 0.001–0.05), and in Urco Miraño enclosed 4 households (6.8% of households) encompassing 9.1% of the community population (LISA range = 0.02–0.60; p-value range = 0.01–0.05). In contrast, only 2 households (10% of households) and 13.1% of inhabitants composed the high-risk cluster in Primero de Enero (LISA range = 0.56–1.35; p-value range = 0.01–0.05). In addition, 4 low-high outliers (meaning that households with low parasitaemia were surrounded by a neighborhood with high parasitaemia) were detected in Gamitanacocha and 2 in Urco Miraño, whilst only 1 high-low outlier (meaning that households with high parasitaemia were surrounded by a neighborhood with low parasitaemia) was detected in Gamitanacocha, using a 550 m neighborhood buffer (detailed in methods section).

### Multilevel analysis for *P. vivax* parasite prevalence along surveys

The likelihood-ratio tests between Poisson regressions with mixed effects and only fixed effects were statistically significant, showing that the multilevel data structure was best fitted by a mixed-effect regression. The estimates of the random effects coefficients for null, multivariate multi-community model, and community-specific models are presented in Supplementary Tables S2 and S3, showing a moderate clustering of *P*. *vivax* parasitaemia at household level.

The presence of fever at time of visit and primary school attendance were significant in the bivariate multi-community analysis (Table 3). In addition, the *P*. *vivax* parasite prevalence was higher in Libertad (prevalence ratio – PR = 1.72; 95% CI = 1.14;2.58; p-value = 0.01) and Urco Miraño (PR = 1.54; 95% CI = 1.03;2.31; p-value = 0.04), than in Gamitanacocha. Other epidemiological variables possibly associated (p-value < 0.1) with higher *P*. *vivax* parasite prevalence were observed with the community-specific approach: education level in Gamitanacocha (primary school) and Primero de Enero (secondary and higher); presence of fever at time of visit and livestock in dwelling in Urco Miraño, a travel record, occupation and location in a high-risk cluster in Primero de Enero, and wall material and age between 15 and 39 in Gamitanacocha (Table 3).

**Table 3:** Fixed effects of multi-community and community-specific bivariates models for *P*. *vivax* parasitaemia.

	Multi-community model			Community-specific models											
				Gamitanacocha			Libertad			Primero de Enero			Urco Miraño		
	PR	95% CI	p-value	PR	95% CI	p-value	PR	95% CI	p-value	PR	95% CI	p-value	PR	95% CI	p-value
**Null model**															
Constant	0.18	(0.16–0.20)	<0.001	0.12	(0.08–0.17)	<0.001	0.2	(0.17–0.24)	<0.001	0.14	(0.10–0.19)	<0.001	0.18	(0.15–0.21)	<0.001
**Time-dependent level covariates**															
**Travel history** (**Ref = No**)															
Yes	1.06	(0.76–1.49)	0.714	0.85	(0.26–2.83)	0.796	0.71	(0.37–1.35)	0.3	2.04	(0.99–4.20)	0.052*	1.28	(0.74–2.19)	0.374
**Fever symptom** (**Ref = No**)															
Yes	1.68	(1.09–2.59)	0.018**	1.06	(0.25–4.45)	0.942	1.33	(0.62–2.85)	0.455	—	—	—	2.57	(1.46–4.53)	0.001**
**Individual level covariates**															
**Gender** (**Ref = Female**)															
Male	0.99	(0.81–1.21)	0.937	1.26	(0.58–2.66)	0.572	0.89	(0.65–1.22)	0.471	1.35	(0.70–2.58)	0.365	0.95	(0.70–1.29)	0.753
**Age groups** (**Ref = <15 years**)															
15–39.9	1.18	(0.94–1.49)	0.148*	2.11	(0.91–4.89)	0.082*	1.01	(0.70–1.45)	0.942	1.33	(0.65–2.75)	0.432	1.19	(0.83–1.70)	0.355
≥ 40	0.97	(0.74–1.26)	0.838	1.06	(0.33–3.40)	0.927	0.7	(0.45–1.07)	0.1	1.66	(0.72–3.86)	0.235	1.1	(0.74–1.62)	0.63
**Education level** (**Ref = None**)															
Primary school	1.3	(1.02–1.65)	0.030**	2.89	(0.99–8.042	0.052*	1.14	(0.80–1.63)	0.465	1.54	(0.67–3.59)	0.308	1.33	(0.91–1.94)	0.140*
Secondary school or higher	1.28	(0.94–1.73)	0.116*	1.17	(0.21–6.37)	0.859	1.21	(0.67–2.16)	0.527	2.68	(0.94–7.63)	0.065*	1.24	(0.81–1.89)	0.315
**Occupation** (**Ref = Not a Logger**, **fisher and farmer**)															
Yes	1.16	(0.95–1.42)	0.154*	1.69	(0.77–3.70)	0.195*	0.89	(0.64–1.22)	0.47	1.73	(0.92–3.28)	0.090*	1.28	(0.94–1.76)	0.120*
**Household level covariates**															
**High-risk cluster** (**Ref = No**)															
Yes	1.1	(0.88–1.34)	0.412	1.97	(0.68–5.70)	0.21	—	—	—	2.09	(1.09–4.00)	0.027**	1.24	(0.90–1.71)	0.192*
**Household structure** (**Ref = Open**)															
At least one closed room	0.93	(0.76–1.15)	0.527	1.16	(0.52–2.60)	0.719	0.8	(0.58–1.10)	0.182*	0.83	(0.43–1.59)	0.585	0.99	(0.71–1.39)	0.952
**Wall material** (**Ref = Cement or wood**)															
Palm, leaf, straw, corrugated plastic	1.06	(0.83–1.35)	0.651	2.08	(0.93–4.62)	0.073*	0.9	(0.57–1.41)	0.644	0.82	(0.36–1.87)	0.638	1.13	(0.79–1.61)	0.519
**Roof material** (**Ref = Corrugated plastic**)															
Palm, leaf, straw	1.1	(0.86–1.41)	0.438	0.56	(0.26–1.22)	0.147*	1.14	(0.50–2.58)	0.752	—	—	—	1.21	(0.88–1.67)	0.24
**Household sprayed** (**Ref = Yes**)															
No	0.91	(0.58–1.44)	0.71	1.89	(0.76–4.69)	0.172*	0.83	(0.45–1.54)	0.562	0.54	(0.13–2.25)	0.399	1.84	(0.25–13.47)	0.548
**Electricity supply** (**Ref = No**)															
Yes	0.96	(0.79–1.18)	0.764	0.63	(0.25–1.56)	0.319	1.13	(0.80–1.60)	0.488	0.77	(0.38–1.55)	0.467	0.88	(0.59–1.31)	0.543
**Livestock** (**Ref = Yes**)															
No	1.1	(0.88–1.36)	0.4	0.95	(0.38–2.36)	0.907	0.99	(0.71–1.37)	0.932	0.92	(0.49–1.74)	0.803	1.35	(0.95–1.91)	0.094*

Mixed-effect Poisson Models; PR = Prevalence Ratio; Wald test p-value; *p-value < 0.2; **p-value < 0.05.

The estimates of the multivariate multi-community model are presented in Table 4. The presence of fever at time of visit (adjusted prevalence ratio – APR = 1.73; 95% CI = 1.11;2.71; p-value = 0.01), primary school attendance (APR = 1.27; 95%CI = 1.01;1.60; p-value = 0.04), and living in a household located in a high-risk cluster (APR = 1.47; 95% CI = 1.09;1.98; p-value = 0.01), remained associated with higher *P*. *vivax* parasite prevalence, while age >40 years (APR = 0.69; 95% CI = 0.49;0.98; p-value = 0.04) remained associated with a lower *P*. *vivax* parasite prevalence, even after adjusting for community. Furthermore, significant interaction terms demonstrated that the effects of travel, and occupation on *P*. *vivax* parasite prevalence varied by community. The *P*. *vivax* parasite prevalence was higher in loggers, fishermen and farmers from Urco Miraño (APR = 1.52; 95% CI = 1.02;2.27; p-value = 0.04), and Gamitanacocha (APR = 2.07; 95% CI = 1.00;4.28; p-value = 0.05) than those from Libertad. Whereas travel in a period of a month was associated with higher *P*. *vivax* parasite prevalence only in Primero de Enero (APR = 2.93; 95% CI = 1.13;7.64; p-value = 0.03) (Table 4). Other interactions terms were tested (i.e. fever and age) but none were significant.

**Table 4:** Fixed effects of multi-community multivariate model for *P*. *vivax* parasitaemia.

	APR	95% CI	p-value
**Community** (**Ref = Libertad**)			
Urco Miraño	0.58	(0.38–0.88)	0.007**
Primero de Enero	0.4	(0.20–0.81)	0.003**
Gamitanacocha	0.29	(0.11–0.76)	0.003**
**Travel history last month** (**Ref = No**)			
Yes	0.71	(0.44–1.15)	0.101*
Travel*community			
Yes*Urco Miraño	1.63	(0.87–3.08)	0.090*
Yes*Primero de Enero	2.93	(1.13–7.64)	0.028**
Yes*Gamitanacocha	0.93	(0.35–2.44)	0.267
**Fever symptom** (**Ref = No**)			
Yes	1.73	(1.11–2.71)	0.013**
Age groups (Ref = <15 years)			
15–39.9	0.85	(0.66–1.10)	0.119*
≥ 40	0.69	(0.49–0.98)	0.041**
**Education level** (**Ref = None**)			
Primary school	1.27	(1.01–1.60)	0.040**
Secondary school or higher	1.27	(0.96–1.68)	0.074*
**Occupation** (**Ref = Not a Logger**, **fisher and farmer**)			
Yes	1.04	(0.79–1.39)	0.256
Occupation*community			
Yes*Urco Miraño	1.52	(1.02–2.27)	0.041**
Yes*Primero de Enero	1.73	(0.95–3.15)	0.064*
Yes*Gamitanacocha	2.07	(1.00–4.28)	0.050**
**High-risk cluster location** (**Ref = No**)			
Yes	1.47	(1.09–1.98)	0.011**

Mixed-effect Poisson Models; APR = Adjusted Prevalence Ratio; p-values and 95% CI adjusted by Adaptive False Discovery Rate (AFDR); *p-value < 0.2; **p-value < 0.05.

The multivariate community-specific models (Table 5) show remarkable differences across communities. Three factors were associated with higher *P*. *vivax* parasite prevalence in Gamitanacocha: loggers, fishermen and farmers (APR = 2.21; 95% CI = 1.01:4.84; p-value = 0.04) living in HHs with walls made of palm, leaf, straw or corrugated plastic (APR = 3.11; 95% CI = 1.27:7.61; p-value = 0.01), which had not been treated with indoor residual spraying (APR = 2.58; 95% CI = 1.04:6.42; p-value = 0.04) after adjusting for whether HH is located in a high-risk cluster. In Libertad, no significant variables were retained in the multivariate model, showing a predominant effect of age over 40 years (APR = 0.71; 95% CI = 0.50:1.02; p-value = 0.06) related with lower *P*. *vivax* parasite prevalence after adjusting for household structure.

**Table 5:** Fixed effects of community-specific multivariate models for *P*. *vivax* parasitaemia.

	Gamitanacocha			Libertad			Primero de Enero			Urco Miraño		
	APR	95% CI	p-value	APR	95% CI	p-value	APR	95% CI	p-value	APR	95% CI	p-value
**Travel history last month** (**Ref = No**)												
Yes							3.11	(1.25–7.74)	0.011**			
**Fever symptom** (**Ref = No**)												
Yes										2.64	(1.31–5.31)	0.002**
**Gender** (**Ref = Female**)												
Male							1.56	(0.93–2.61)	0.074*			
**Age groups** (**Ref = **<**15 years**)												
15–39.9				1.09	(0.84–1.41)	0.196*						
≥40				0.71	(0.50–1.02)	0.063*						
**Occupation** (**Ref = Not a Logger**, **Fisher and Farmer**)												
Yes	2.21	(1.01–4.84)	0.040**				1.43	(0.88–2.31)	0.096*	1.36	(1.01–1.82)	0.040**
**High-risk cluster location** (**Ref = No**)												
Yes	2.22	(0.91–5.38)	0.069*				2.96	(1.26–6.90)	0.009**	1.24	(0.96–1.58)	0.076*
**Household structure** (**Ref = Open**)												
At least one closed room				0.81	(0.63–1.04)	0.076*						
**Wall material** (**Ref = cement/wood**)												
Palm, leaf, straw, corrugated plastic	3.11	(1.27–7.61)	0.013**									
**Household sprayed** (**Ref = Yes**)												
No	2.58	(1.04–6.42)	0.040**									

Mixed-effect Poisson Models; APR = Adjusted Prevalence Ratio; p-values and 95% CI adjusted by Adaptive False Discovery Rate (AFDR); *p-value < 0.2; **p-value < 0.05.

In Primero de Enero inhabitants that reported travel in the last month (APR = 3.11; 95% CI = 1.25:7.74; p-value = 0.01), and living in a high-risk cluster (APR = 2.96; 95% CI = 1.26:6.90; p-value = 0.01) were associated with higher *P*. *vivax* parasite prevalence after adjusting for occupation and gender. Finally, in Urco Miraño, the presence of fever at time of visit was the most important variable (APR = 2.64; 95% CI = 1.31:5.31; p-value = 0.002) associated with a higher *P*. *vivax* parasite prevalence after adjusting for occupation (APR = 1.36; 95% CI = 1.01:1.82; p-value = 0.04), and whether HH is located in a high-risk cluster (APR = 1.24; 95% CI = 0.96:1.58; p-value = 0.08).

## Discussion

In the Peruvian Amazon, as in other malaria endemic regions, micro-geographical variation in malaria parasitaemia risk factors are consistently under-appreciated by public health policy makers as important for malaria control strategies. Using molecular tools and active case detection, this study demonstrated that proportion of *P*. *vivax* parasitaemia was highly heterogeneous both within and among riverine Amazonian communities where a large proportion of inhabitants carried sub-microscopic *P*. *vivax* and *P*. *falciparum* parasitaemia^14, 33, 48–51^. This study further found that communities at small-to-moderate spatial scales (1 to 15 km) differed in *P*. *vivax* parasite prevalence, and that such differences were influenced by factors such as age, educational attainment, and location of households within high-risk clusters, as well as factors that were tied to a local micro-geographic context, such as travel and occupation. We observed that spatial clustering patterns of *P*. *vivax* parasitaemia differed in communities with different occupation-related activity profiles. The complex transmission patterns observed in this area seem to be determined by human movement among communities in the same micro-basin (i.e. tributary streams network that flows into the Amazon River). This concept is key yet neglected and must be understood to be a critical intervention point for malaria control in the Peruvian Amazon, a concept generalizable in similar contexts elsewhere.

The magnitude of asymptomatic parasitaemia in this region challenges the traditional definition of hypoendemicity with regard to transmission from humans to mosquitoes. Previous studies indicate that only a few antecedent infections—whether with *P*. *vivax* or *P*. *falciparum* in Amazonia—can lead to clinical immunity in this region that is manifested by asymptomatic parasitaemia^13, 33, 49, 52, 53^. Our data suggest that the relatively high prevalence of malaria parasitaemia in the study populations leads to the maintenance of transmission on a micro-geographic scale. The differences between the age-stratified proportions of *P*. *vivax* and *P*. *falciparum* parasitaemias detected by qPCR and light microscopy seems to be the result of a combination of exposure and immunity as observed elsewhere^14, 54, 55^. One unavoidable issue with the present analysis is that we cannot differentiate the contribution of acute, new infections from hypnozoite relapses from a previous mosquito bite to transmission quantitatively. However, previous studies indicate that in the Peruvian Amazon, most parasitaemias originated from relapses are asymptomatic and hence capable of transmission to mosquitoes^43, 56^. Furthermore, in tropical regions such as the Peruvian Amazon, relapses tend to occur rapidly—at a frequency of three to ten weeks^56–60^. In any event, our data are consistent with the notion that asymptomatic *P*. *vivax* parasitaemia —whether from new or distant infection— leads to maintenance of malaria transmission on a scale larger than previously known^13, 14, 33^. One limitation of this analysis is the actual demonstration and quantification of the transmissibility of asymptomatic, low-level parasitaemia to mosquitoes; such work is in progress in our study sites/laboratory in Loreto.

The comprehensive spatio-temporal follow-up data allowed for a detailed assessment of *P*. *vivax* parasitaemia in these communities, which enabled us to properly understand the transmission potential of patent/sub-patent, and symptomatic/asymptomatic malaria parasitaemia, regardless if it was a new acute infection or a relapse from hypnozoites. If exposure and infection are indeed highest over small geographical areas, most inhabitants will harbor low parasitaemia —likely due to host acquired immunity— with important implications for malaria control strategies^25–29, 61, 62^, especially if inhabitants frequently come into contact with the high transmission environments as observed in other studies^14, 20, 33^. Our within-community spatial clustering analysis shows that communities such as Gamitanacocha and Urco Miraño, which were found to have larger clusters of *P*. *vivax* parasitaemia, were identified as closed and isolated communities. Indeed, the data showed that nearly all households had at least one asymptomatically infected individual; suggesting that at larger scale (i.e. micro-basin), these communities are foci of parasites. Nonetheless, community level spatial clustering may be a consequence of the spatial distribution of dwellings and mosquito breeding sites. As previously demonstrated, *An*. *darlingi* has adapted to a diverse habitats^63, 64^, with focal activity related to high density nearby breeding sites^65, 66^. In tropical riverine communities, such favourable conditions are shaped by seasonal flooding and intermittent heavy rains^67, 68^, allowing multiple breeding sites mainly in the large number of slow-flow streams proximate to households^69–71^. In contrast, communities with higher occupational-related population movement, like Libertad and Primero de Enero, had smaller or non-clustering of parasitaemia.

In Urco Miraño, the high-risk clusters were observed in a highly deforested area with substantial surrounding vertical vegetation. Studies have shown that such deforested areas are preferred breeding sites for *An*. *darlingi* in rural Amazon^72–74^. Urco Miraño is a geographically isolated community on the Napo River, and is the settlement of the Yagua ethnic group^75^. Traditional lifestyle and customs may play a role in community interactions; thus, population movement was found to be less common than communities in the Mazan River micro-basin. The population movement in this community, mainly towards Mazan community and the city of Iquitos, seems to be shaped by work activities related to visitor’s ecological tourism activities as in other communities of the Napo River micro-basin. A similar clustering pattern was observed in one of the most distant communities in the Mazan River micro-basin, Gamitanacocha. In this community the high-risk clusters were located near the ‘*cocha*‘ (lagoon), where the stagnant water ecosystem likely contributes to suitable *An*. *darlingi* breeding sites. Consistently, in Gamitanacocha, a high population movement was observed towards Maucallacta, a closer community with low risk of malaria during the study period according to RHDL records.

The remaining communities in the Mazan River micro-basin showed a different spatial clustering of malaria parasitaemia and population movement patterns. Libertad and Primero de Enero are contiguous communities with the highest malaria transmission in the Mazan River micro-basin; nevertheless, both are the biggest and most developed communities of this micro-basin. These communities are mainly devoted to extractive activities, and thus, the occupational-related mobility was higher than in Gamitanacocha or Urco Miraño. Interestingly, the occupational-related localities reported by Parker *et al*.^44^ as hyperendemic malaria transmission areas, were the most important destinations of occupational-related travels by inhabitants of Libertad and Primero de Enero. This liability to the frequent influx of infected individuals suggests a high vulnerability (i.e. malaria importation rate) in these communities^76, 77^, as in other Amazonian Regions^78^.

Our findings show that *P*. *vivax* predominated over *P*. *falciparum*, consistent with RHDL routine surveillance data. In addition to communities in different micro-basins showing a high heterogeneity of *P*. *vivax* parasite prevalence, significant differences were observed between the neighbouring communities of Libertad and Primero de Enero. Our findings in riverine communities from the Amazon region are consistent with recent studies that showed a high heterogeneity between villages at a micro-geographical scale along the China-Myanmar border, where *P*. *vivax* predominated^79, 80^; in western Kenya, where most *P*. *falciparum* infections were sub-microscopic and asymptomatic^81^; and in peri-urban communities in the Loreto Region of Peru^14^. In addition to human population movement, vector populations and behaivours might be other contributing factors to the observed micro-geographical heterogeneities in malaria tranmission. Moreno *et al*.^10^ reported a high spatio-temporal heterogeneity in the entomological inoculation rate (EIR) among riverine and semi-urban communities in the Amazon Region; and Lainhart *et al*.^9^ reported two major *An*. *darlingi* subpopulations among peri-Iquitos communities, including Mazan samples collected in Libertad. Those genetically differentiated populations might be associated with different *An*. *darlingi* biting behaviors^82^.

The multilevel analysis provided important insights about malaria transmission potential in these micro-basins. Across all communities (i.e. multi-community model), our findings showed that young inhabitants had higher *P*. *vivax* parasite prevalence. This age-dependent parasitaemia effect is consistent in general with studies carried out in Kenya^81, 83^, Papua New Guinea^54^, Botswana^84^, Ethiopia^85, 86^, and Brazil^48^, where parasite prevalence and density decreased with age. Consistently, the presence of fever at time of visit remained a specific symptom for *P*. *vivax* parasite prevalence particularly in young children, indicating a positive relationship between age and acquired immunity. In the Peruvian Amazon region, individuals’ control of parasite density may be fostered by the high clonality of parasite populations observed at a local level in the Peruvian Amazon Region^8, 32, 34, 87^, and the high EIR, as described in similar communities upriver in the Mazan River micro-basin^44^.

Importantly, the *P*. *vivax* parasite prevalence in Libertad was higher than in other communities from the same micro-basin or the Napo River micro-basin, taking into account the within-community heterogeneity, thus confirming the high heterogeneity between communities. In addition, heterogeneity in risk factors for *P*. *vivax* parasite prevalence was observed between communities. The significance of the interaction terms of community versus travel and occupation indicates that the communities did not behave as a single population. Instead, these results suggest that these risk factors seems to be tied to a local micro-geographic context. These risk factors for malaria parasitaemia were previously reported in the Amazon Region^14, 61, 88^. However, this study demonstrates a community-specific effect of these variables. This spatial non-stationarity association between *P*. *vivax* parasitaemia and risk factors was observed in several scenarios where a global risk factor estimate can become inappropriate since they averaged relationships that are not transferable to all local contexts^89–92^.

Using the community-specific approach, we observed particular risk factors for each community, such as occupation in Gamitanacocha and Urco Miraño. Interestingly, the presence of fever at time of visit was associated with higher *P*. *vivax* parasite prevalence only in Urco Miraño. This clinical manifestation was typically observed in susceptible populations not previously exposed to current parasite populations^93, 94^, suggesting a high receptivity (i.e. the potential for ongoing local transmission) in this community. A further factor associated with *P*. *vivax* parasite prevalence in Gamitanacocha using this approach was that inhabitants who reported that their households had not received IRS by the MOH had higher *P*. *vivax* parasite prevalence. In 2014, RHDL started an IRS campaign in several communities along the Mazan and Napo Rivers, including all communities in our study area. This protective effect of IRS has also been reported in previous studies^47, 95^ and is recommended as an important component of control and elimination programs^96, 97^. Nevertheless, this effect was not significant in other communities, supporting the hypothesis that mobile individuals acquire infection outside their residence communities.

The intense flow of infected and non-infected people between communities of these micro-basins, plus introductions due to population movement to location outside the study area, has likely established a tangled yet open transmission network, generating complex community transmission patterns^98, 99^, that over space and time would be associated with increased heterogeneity of transmission^18, 100, 101^, facilitating parasite population admixture^30–32^. Delgado-Ratto *et al*.^34^ showed the importance of human population movement on the geographic distribution of parasites and consequently the development of sub-structured parasite populations observed in peri-Iquitos communities. The genotyping analysis of isolates from the Mazan community reported by Van den Eede *et al*.^32^ showed high heterogeneity and strong differentiation from other peri-Iquitos isolates, suggesting multiple geographic sources of parasite sub-populations. Activation of heterologous hypnozoites acquired over the lifetime of the adult population^39, 102^ could not be excluded as an explanation for the sub-structured parasite population observed in this communities. However, relapses in these areas presumably arose from infections in the near past (3–10 weeks)^57–60^, suggesting that the origin of the parasite population admixture is mainly the human population movement between communities with clustered transmission. As this riverine context is common in Amazonia, it is likely that the malaria rebound observed since 2011 was accelerated based on the high vulnerability and receptivity of riverine communities^15, 77, 103, 104^. Thus, further work is needed to determine ‘source’ and ‘sink’ areas for malaria parasites in the Amazonian riverine network^105–107^ and to develop a comprehensive framework of human population movement in Amazonian communities^108, 109^ to prevent malaria importation during transit (from hypo- to hyper- endemic areas) or upon return^100^.

This study has some limitations. Despite efforts in each survey to include all community members, we achieved a rate of at least 80% participation at each survey point. When we were able to contact a person, we did not observe any refusal to participate. Instead, the most important reason for incomplete or missing data was the fact that individuals were away from the area for different reasons, such as work, national army recruitment, and festivities. We used generalized linear mixed models which are valid under the assumption of missingness at random (MAR)^110, 111^, and believe this assumption to be appropriate since all factors in our dataset associated with missingness were included in the final models, and did not show a relevant effect (>10% change) on the risk factor estimates. We thus opted for the most parsimonious models, as presented in Tables 4 and 5. Note that we included mobility (due to work, national army recruitment, festivities) and drivers to different malaria-transmission environments, as potential confounders to our risk factor estimates. Another issue that might have affected our estimates is the limit of detection of our molecular diagnostic method that uses filter papers. As previously reported in Amazonian settings^61^, infected individuals can harbor very low parasitaemia, and at those parasite densities the probability of detecting a malaria parasite DNA molecule is random. That being said, PCR based on filter papers only underestimates the potential effect of asymptomatic parasitaemia on parasite mobility and transmission, and, as discussed above, the transmission potential of extremely low parasitaemia remains to be determined.

In conclusion, this study highlights that to tailor strategies for effective malaria control and future elimination in riverine communities of the Amazon Region, it will be essential to take local geography and human socio-demographics into account to estimate dynamics of ongoing malaria transmission. Evidence from this and other studies regarding the large reservoir of sub-microscopic infections, high heterogeneity in risk factors for *P*. *vivax*, and spatial clustering of transmission, strongly suggests that public health authorities should reformulate malaria control strategies based on sensitive parasite detection and active surveillance. Larger, comprehensive assessments are needed to advance the understanding of the impact of human mobility on malaria transmission in riverine communities before decisions of mass drug administration on a community or population level can be made, for example issues of community acceptability and which drugs to use and their safety.

## Methods

### Ethics Statement

The study was approved by the Ethics Review Board of the Universidad Peruana Cayetano Heredia in Lima and the Regional Health Direction of Loreto. Prior to study enrollment, every adult participant signed an informed consent and every participant under 18 years old provided informed assent with parental or guardian written approval. All the methods were carried out in accordance with the approved guidelines.

### Study area and population

The present study was conducted in the district of Mazan (North of Iquitos), province of Maynas in Loreto department. The population is mainly mestizo (i.e. mixed-race identity), very poor, and lives in semi-open wooden houses without screens. The local economy depends on agriculture, lumber, and fish extraction. The weather is tropical, with minimum temperatures of 17 °C to 20 °C in the months from December to March, and maximum temperatures up to 36 °C in June and July. The average humidity is 84% with heavy rains from November to May^5, 67^. Mazan district is comprised of several small communities, and the capital and largest community is the community of Mazan (3.503° S, 73.094° W), located near the confluence of the Mazan and Napo Rivers, about 55–60 km from the city of Iquitos (1 hour by boat).

Malaria transmission in Mazan district has an unstable and seasonal epidemiological pattern, with a peak between May and September. The primary vector is *An*. *darlingi*, highly anthropophilic^10^ and about 85% of malaria cases reported by the RHDL are caused by *P*. *vivax* (the remainder being exclusively due to *P*. *falciparum*) distributed across all ages. Mazan is considered a very high-risk district for malaria transmission; 1,954 cases were reported in 2014, indicating an annual parasite index (API) of 141.1 cases per 1,000 inhabitants.

### Study design and selection of communities

In March 2015, a comprehensive census and household geo-referencing was conducted in ten high-risk malaria communities (according to RHDL historical data) in Mazan district. From March to April 2015, these communities were under weekly surveillance to detect communities where any unusual increase of cases might occur. Surveillance was based on monitoring the slide positivity rate (SPR) from passive case detection (PCD) of the RHDL. Study communities were selected based on two criteria: a) the proportion of the population screened for malaria (by thick or thin blood films) was larger than 20% in the previous 8 weeks, and b) the SPR of the previous 8 weeks remained above 5% for two consecutive weeks. These criteria resulted in the selection of four communities (Fig. 1): Gamitanacocha (3.426° S, 73.318° W), Libertad (3.496° S, 73.234° W), Primero de Enero (3.494° S, 73.221° W), and Urco Miraño (3.361° S, 73.064° W).

Subsequently, a population-based multi-level cohort study was conducted on the selected communities. From May to July 2015, four consecutive cross-sectional active case detection (ACD) surveys rounds were conducted in each of the four communities, one ACD survey every 10 days. To look for risk factors and heterogeneities in risk factors for *P*. *vivax* parasitaemia between communities taking into account different levels of *P*. *vivax* parasitaemia clustering, a spatial and multilevel analysis was carried out.

### Data collection

In the census, each household in the communities was encoded (6-digit numeric code) and geo-referenced using a Global Positioning System (GPS) hand device (Garmin’s GPSMAP 60CSx, Garmin International Inc., USA). All members of each household were invited to participate, and, upon consent, a unique 8-digit numeric code was assigned to each participant. A structured questionnaire designed and previously pilot-tested was used to collect individual and household data (age, gender, socioeconomic characteristics, recreation and occupational activities, malaria history, use of preventive measures, ownership of animals, structural characteristics of the house, household services, etc.).

In each ACD survey, data regarding socio-demographic characteristics and clinical manifestations at time of visit were collected. Each participant was clinically examined for fever or any other malaria symptom. Blood samples were collected on every household member (regardless of symptoms) by finger-prick for microscopic examination (thick and thin blood films), and for PCR testing (blood spot on filter paper - Whatman grade 3, Whatman, Springfield Mill, USA). Blood samples were labeled during fieldwork with pre-printed labels with 9-digit numerical codes. This code was used in the fieldwork and at the Institute of Tropical Medicine “Alexander von Humboldt”, (ITM-AvH) in Lima, where samples were processed. New participants were enrolled during the ACD surveys. Participants with malaria diagnosis by microscopy at ACD surveys were treated following national guidelines.

During the census and the ACD surveys, houses were visited up to 3 times in a period of 3 days to maximize subject participation. Data were collected using Open Data Kit (ODK)^112, 113^ on mobile devices without network connection. Data were synchronized with the project´s server in Lima, and linked to laboratory results using the 9-digit unique numerical codes.

### Laboratory procedures

#### Microscopy

Microscopy examination was performed the same day of sample collection in each ACD survey. Thick and thin blood smears were stained with 10% Giemsa solution for 10 minutes and were examined to determine the parasite density by counting the number of parasites on asexual stage and gametocytes by 200 leucocytes (L) and assuming a concentration 8,000 L/μl of blood. A sample was considered negative if no malaria parasite was detected after the examination of 100 fields of microscopy^114^. Quality control was done blindly on all positive slides and 10% of randomly chosen negative slides by a senior technician at ITM-AvH.

#### Real Time Quantitative PCR (qPCR)

Filter paper areas containing blood were cut into ~6 mm^2^ sections for the parasite genomic DNA extraction using E.Z.N.A.® Blood DNA Kit (Omega Bio-tek®, USA), following manufacturer instructions with slight modifications – addition of TEN (20 mM Tris-HCl, pH 8.0; 2 mM EDTA, pH 8.0; 0.2 M NaCl) buffer, supplemented with SDS 10% w/v – and stored at 4 °C for immediate use and at −20 °C for further analysis.

Quantitative real-time polymerase chain reaction (qPCR) testing was done following a modified protocol from Mangold *et al*.^115^ using PerfeCta SYBR Green Fast Mix (Quanta Biosciences, MD, USA). Briefly, this method amplifies the 18SSU rRNA gene sequence of the *Plasmodium* species-specific region. After the amplification, analysis of the differences in melting curves provided an accurate differentiation between *Plasmodium* species.

#### Spatial Analysis

To assess whether spatial autocorrelation of *P*. *vivax* parasitaemia occurred in the study area, a global Moran’s *I* statistics^116^ was computed to describe the overall spatial dependence of *P*. *vivax* parasitaemia in each community and Anselin’s Local Moran’s *I* (which is one type of Local Indicator of Spatial Association - LISA) was performed with a correction for multiple and dependent tests using the false discovery rate (FDR)^117, 118^ to identify local patterns and high-risk areas. These analyses were performed with the proportion of individuals with *P*. *vivax* parasitaemia at household level as outcome. The time-series data for each participant were collapsed to a single register defined as at least one *P*. *vivax* parasitaemia (positive result by qPCR) of the 4 ACD surveys. For the spatial data processing and visualization, QGIS 2.16 (QGIS Development Team, 2016. QGIS Geographic Information System. Open Source Geospatial Foundation Project) was used.

Distance weighted neighborhoods were defined in GeoDa v1.8 (Luc Anselin, 2016, USA)^119^. Two factors guided the criterion for choosing distance threshold/band. The first is the flight range of mosquitoes in the Amazon region. The longest reported flight range of *An*. *darlingi* in the Brazilian Amazon is 7.2 km^120^, but most mosquitoes fly within 400–500 m^65, 66^. Second, in these communities, buffers larger than 2,000 m cover the entire community. A non-parametric spatial autocorrelation analysis was performed in GeoDa to evaluate neighborhood distance bands from 100 m to 2,000 m. As result, a band of 550 m displays the maximum level of spatial autocorrelation and ensure that each household has a neighbor. With this distance band the global Moran’s *I* was used to test the null hypothesis that measured values at one household location are independent of values at other households locations, and LISA analyses identify significant relations between the household values and the neighborhood-lagged values in each community as: high-risk clusters (high-high) where both proportion in household and neighborhood were high, low-risk clusters (low-low) where both proportion in household and neighborhood were low, and outliers (high-low or low-high) where the proportion in household and neighborhood were different.

### Statistical Analysis

The multilevel design of the study consisted of repeated observations of individuals (up to 4 ACD surveys), nested within households, nested in the four selected communities (Supplementary Fig. S1). A generalized linear mixed-effects model (GLMM), with fixed effects and a random intercept estimation for each level was used to control for clustered sampled data. Due to the inability to distinguish between recrudescent, latent or new infections, we only used the first *P*. *vivax* parasitaemia as the dependent variable in the models, and the subsequent observations were excluded from the analysis. We modeled the presence of *P*. *vivax* parasitaemia (i.e. parasite prevalence) regardless of symptoms and/or previous treatment. All *P*. *falciparum* parasitaemia were excluded from the analysis. Poisson regression models were used to estimate the prevalence ratio (PR) of the outcome, assuming a binomial distribution for the error term, and a log link function^121–123^.

Covariates within each level were included in the analysis: 7 at the household-level: whether household is located inside a high-risk cluster (determined by LISA) or its 550m-neighborhood buffer, household structure (houses with at least one closed room or not), walls material, roof material, whether household received indoor residual spraying (IRS) by the RHDL, had electricity supply, and had livestock inside dwelling; 4 at the individual-level: subjects’ gender, age (categorized as <15 years, and ≥15 years for descriptive purpose and <15 years, between 15 and 39.9 years, and ≥40 years for regression analysis), education level (categorized as none or primary school, and secondary school or higher for descriptive purpose and none, primary school, and secondary school or higher for regression analysis), and outdoor occupation (logger, fisher and farmer; and others), and 2 time-dependent variables: travel history in the last month, and fever symptom at each survey round (Supplementary Fig. S1).

To evaluate if factors associated with *P*. *vivax* parasitaemia vary at micro-geographical level (micro-epidemiological factors), two approaches were used: The first consisted in the construction of a single model including all communities and exploring interaction terms between each factor and community (from here on termed “multi-community” model), and in the second approach, separate regression models were built for each community (from here on termed “community-specific” models). Therefore, three levels (observation, individual, and household) were included in the equation for our multilevel model of the dependent variable *P*. *vivax* parasitaemia (*y*_*ijk*_):

1 $$\mathrm{log}[E({y}_{ijk})]={\delta }_{000}+({V}_{00k}+{U}_{0jk})+{\beta }_{1ijk}{x}_{1ijk}+\ldots +{\varepsilon }_{ijk},\quad y \sim Poisson$$

where the *ijk* subscript refers to the *i* ^th^ observation of the *j* ^th^ individual from the *k* ^th^ household; *δ*_000_ represents the fixed effect of the intercept and (*V*_00*k*_ + *U*_0*jk*_) represents the intercept random effects of households and individuals within households, respectively; *β*_1*jk*_ represents the fixed effects of the slope for the covariates terms, and *ε*_*ijk*_ refers to the random error term.

All data analyses were done in STATA 14 (StataCorp. 2015. Stata Statistical Software: Release 14. College Station, TX). Statistical significance was defined at p-value < 0.05 and 95% confidence intervals (CI) were estimated as appropriate. As in other epidemiological studies that applied multilevel models^61, 124–126^, the bivariate and multivariate analyses were performed using the same multilevel structure. In the bivariate multilevel analysis, the relationship between each factor in all levels (time-dependent, individual and household) and time-dependent *P*. *vivax* parasitaemia was analyzed for the multi-community model, and the community-specific models.

Construction of the multivariate multilevel models was performed using a forward stepwise process, and a likelihood ratio test (LRT) to compare nested models. Multi-community model was constructed adding factors ordered according to the log likelihood in the bivariate model and retained if they had a p-value < 0.2 in the adjusted model. Systematically, interaction terms between each factor in the final model and community were evaluated, and significant (p-value < 0.05) interactions were retained. Community-specific model construction consisted of a stand-alone process for each community. Factors with a p-value < 0.2 in the bivariate model for the corresponding community were introduced and retained if they had a p-value < 0.2 in the adjusted model. A correction for multiple and dependent tests was done with the adaptive false discovery rate (AFDR)^127^, using the ‘MuToss’ package^128^ in R software v3.2 (R Development Core Team, R Foundation for Statistical Computing, Austria).

## Electronic supplementary material

Supplementary information
