# Supplementary material for: Micro-epidemiology and spatial heterogeneity of P. vivax parasitaemia in riverine communities of the Peruvian Amazon: A multilevel analysis
Source: Sci Rep. 2017 Aug 14;7:8082. doi: 10.1038/s41598-017-07818-0 (PMC5556029; doi:10.1038/s41598-017-07818-0)
Supplement: Supplementary file 1 — Supplementary information [file 41598_2017_7818_MOESM1_ESM.pdf]

Micro-epidemiology and spatial heterogeneity of *P. vivax* parasitaemia in riverine communities of the Peruvian Amazon: A multilevel analysis

Carrasco-Escobar Gabriel[1,2,O], Gamboa Dionicia[1,3,4], Castro Marcia C.[5], Bangdiwala Shrikant I.[6,7], Rodriguez Hugo[8], Contreras-Mancilla Juan[1], Alava Freddy[8], Speybroeck Niko[9], Lescano Andres G.[2], Vinetz Joseph M.[3,4,10], Rosas-Aguirre Angel[3,9,*], Llanos-Cuentas Alejandro[2,3,O,*]

1. Laboratorio ICEMR-Amazonia, Laboratorios de Investigación y Desarrollo, Facultad de Ciencias y Filosofia, Universidad Peruana Cayetano Heredia, Lima 31, Peru;
2. Facultad de Salud Pública y Administración, Universidad Peruana Cayetano Heredia, Lima 31, Perú;
3. Instituto de Medicina Tropical "Alexander von Humboldt", Universidad Peruana Cayetano Heredia, Lima 31, Perú;
4. Departamento de Ciencias Celulares y Moleculares, Facultad de Ciencias y Filosofia, Universidad Peruana Cayetano Heredia, Lima 31, Peru;
5. Department of Global Health and Population, Harvard T.H. Chan School of Public Health, 665 Huntington Avenue, Building I, Room 1113, Boston, MA, USA;
6. Department of Biostatistics, University of North Carolina Gillings School of Global Public Health, Chapel Hill, NC, USA;
7. Department of Health Research Methods, Evidence and Impact, McMaster University, Hamilton, ON, Canada;
8. Región de Salud Loreto, Iquitos, Loreto 160, Peru;
9. Research Institute of Health and Society (IRSS), Université Catholique de Louvain, Brussels 1200, Belgium;
10. Division of Infectious Diseases, Department of Medicine, University of California San Diego School of Medicine, La Jolla, California, USA;

[O] Corresponding Authors

Alejandro Llanos-Cuentas

E-mail: alejandro.llanos.c@upch.pe

Gabriel Carrasco-Escobar

E-mail: Gabriel.carrasco@upch.pe

*** These authors contributed equally to this work.**

Key Words: Malaria, *Plasmodium vivax*, molecular epidemiology, sub-microscopic infections, spatial analysis, multilevel model, high-risk clusters, human mobility, human population movement.

SUPPLEMENTARY TABLES AND FIGURES

Supplementary Table S1. Common destinations of travel records self-reported by inhabitants from Gamitanacocha (GC), Libertad (LI), Primero de Enero (PE), and Urco Miraño (UM).

Communities	GC	LI	PE	UM	Total
Individuals	N=18	N=33	N=20	N=25	N=96
Travels	n=36 (%)	n=84 (%)	n=59 (%)	n=82 (%)	n=261 (%)
**Destinations:**					
Iquitos	2 (5.56)	7 (8.33)	5 (8.47)	47 (57.32)	61 (23.37)
Mazan	3 (8.33)	13 (15.48)	1 (1.69)	12 (14.63)	29 (11.11)
Quebradas ^	10 (27.78)	60 (71.43)	38 (64.41)		108 (41.38)
Yurimaguas		1 (1.19)		4 (4.88)	5 (1.92)
Maucallacta	18 (50.00)				18 (6.90)
Paucarillo	2 (5.56)				2 (0.77)
Pebas	1 (2.78)				1 (0.38)
14 de Julio		2 (2.38)			2 (0.77)
Inganoyata		1 (1.19)			1 (0.38)
Santa Cruz			14 (23.73)		14 (5.36)
Orosa River			1 (1.69)		1 (0.38)
Sucusari				2 (2.44)	2 (0.77)
Indiana				3 (3.66)	3 (1.15)
Palmeras				6 (7.32)	6 (2.30)
Pampa Hermoza				4 (4.88)	4 (1.53)
Santa Teresa				4 (4.88)	4 (1.53)

^ Reported by parker

Supplementary Table S2. Random effects of multi-community null and multivariate model for *P. vivax* parasitaemia.

	Var	95% CI
**Null Model**		
Household	0.0151	(0.00005 - 4.46881)
Individual	0	(-)
**Multi-community Model**		
Household	0	(-)
Individual	0	(-)

Mixed-effect Poisson Models with only random intercepts; Var= Variance estimate by mixed-effects model.

Supplementary Table S3. Random effects of community-specific null and multivariate models for *P. vivax* parasitaemia.

	Gamitanacocha		Libertad		Primero de Enero		Urco Miraño	
	Var	95% CI	Var	95% CI	Var	95% CI	Var	95% CI
**Null models**								
Household	0	(-)	0	(-)	0	(-)	0.0362	(0.153 - 0.2142)
Individual	0	(-)	0	(-)	0	(-)	0	(-)
**Community-Specific models**								
Household	0	(-)	0	(-)	0	(-)	0.0219	(0.0001 - 5.0111)
Individual	0	(-)	0	(-)	0	(-)	0.0000	(-)

Mixed-effect Poisson Models with only random intercepts; Var= Variance estimate by mixed-effects model.

Supplementary Figure S1. Multilevel structure of data collected in the study.

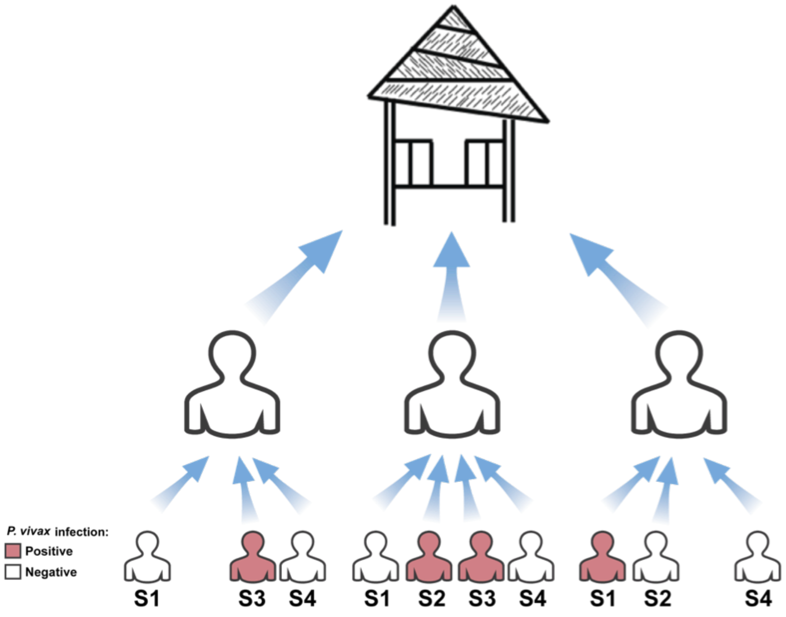

Name of Level	Subscript to index level	Variables measured on each member of the level
Household [154 Households]	*k*	HH structure, walls material, roof material, HH sprayed, electricity supply, livestock and high-risk cluster location.
Individual [820 individuals]	*j*	Gender, age, education, outdoor occupation.
Repeated observations in time [up to 4 surveys (S)]	*i*	Travel history, fever symptom, *P. vivax* parasitaemia by PCR.

HH=Household
